# Melorheostosis: A Case Report on the Atypical Clinical Presentation and Management of a Rare Sclerosing Bone Disease

**DOI:** 10.7759/cureus.112157

**Published:** 2026-07-06

**Authors:** Andrés Felipe Muñoz Leiva, Juan Pablo Aguirre Echeverry, Leobardo Guerrero Beltran, Dolores Cantu Fernandez

**Affiliations:** 1 Orthopaedics and Traumatology, Hospital Juarez de México, Ciudad de México, MEX; 2 Orthopaedics and Traumatology, Hospital Juarez de Mexico, Ciudad de Mexico, MEX; 3 Orthopaedic Surgery, Hospital Juárez de México, Ciudad de México, MEX

**Keywords:** bisphosphonates, bone diseases, melorheostosis, physical therapy modalities, sclerosing

## Abstract

Melorheostosis, also known as Leri disease, is an exceptionally rare mixed sclerosing bone dysplasia of mesodermal origin, with a global prevalence below one per million individuals. It is characterized by progressive cortical and medullary hyperostosis following a sclerotomal distribution, producing the pathognomonic radiographic “dripping candle wax” appearance. We report a 50-year-old woman with no relevant medical background who presented with insidious left lower extremity pain, diffuse limb swelling, local warmth, hyperpigmentation, and reduced range of motion. Radiographs demonstrated eccentric cortical hyperostosis of the left tibia and fibula. Laboratory findings were entirely within normal limits, and tibial biopsy disclosed hyalinized collagen fibers with dystrophic calcifications.

This case is notable for its late-onset presentation and monomelic distribution, contrasting with the early-onset course more commonly described. The patient achieved satisfactory outcomes through a conservative multimodal strategy combining celecoxib and structured physical therapy, with pain declining to 1/10 on the Visual Analogue Scale alongside meaningful functional recovery. This report highlights the importance of integrating clinical, imaging, and histopathological data, and reinforces the value of individualized, multidisciplinary management in this challenging condition.

## Introduction

The National Organization for Rare Disorders (NORD) classifies rare diseases as those affecting fewer than 200,000 individuals. Among these, sclerosing bone dysplasias represent a heterogeneous group of uncommon skeletal disorders arising from disruptions in normal bone development and remodeling pathways [1].

Melorheostosis, commonly referred to as Leri disease, is a mixed sclerosing dysplasia of mesodermal origin that primarily involves cortical bone through hyperostosis and extends to adjacent soft tissues through sclerosis. Its name derives from the Greek terms melos (limb), rhein (flow), and ostosis (bone formation). First characterized in 1922 by Leri and Joanny [2], this condition carries an estimated prevalence of roughly one case per million people, with fewer than 400 publications reported to date. Unlike hereditary dysplasias such as osteopetrosis or osteopoikilosis, melorheostosis occurs sporadically and is not transmitted through conventional inheritance. Its hallmark is the gradual development of longitudinal cortical and medullary hyperostosis following a recognizable sclerotomal distribution [3].

Recent molecular advances have identified somatic mutations in MAP2K1, SMAD3, and LEMD3 genes as key drivers of melorheostosis, with somatic mosaicism accounting for its characteristically asymmetric and segmental distribution. Notably, alterations in the LEMD3 gene and dysregulation of the BMP/TGF-β signaling pathway have been implicated in its pathogenesis, providing a molecular framework that is particularly relevant for readers approaching this condition for the first time.

Herein, we present an atypical late-onset case of monomelic melorheostosis affecting the left tibia and fibula, with the aim of expanding clinical awareness of this rare condition and discussing current diagnostic and therapeutic approaches.

## Case presentation

A 50-year-old woman with no significant medical, pharmacological, or toxicological history presented to the Orthopedic Department reporting progressive, insidious pain in the left lower extremity evolving over several months. She denied any history of trauma, recent infections, or systemic symptoms such as fever or unintentional weight loss.

On physical examination, the left leg exhibited diffuse volumetric enlargement, local warmth to palpation, and irregular hyperpigmentation of the overlying skin. Active range of motion at the ankle and knee was substantially diminished, a finding that was confirmed on passive examination. Neurovascular assessment of the distal extremity was intact.

**Figure 1:**
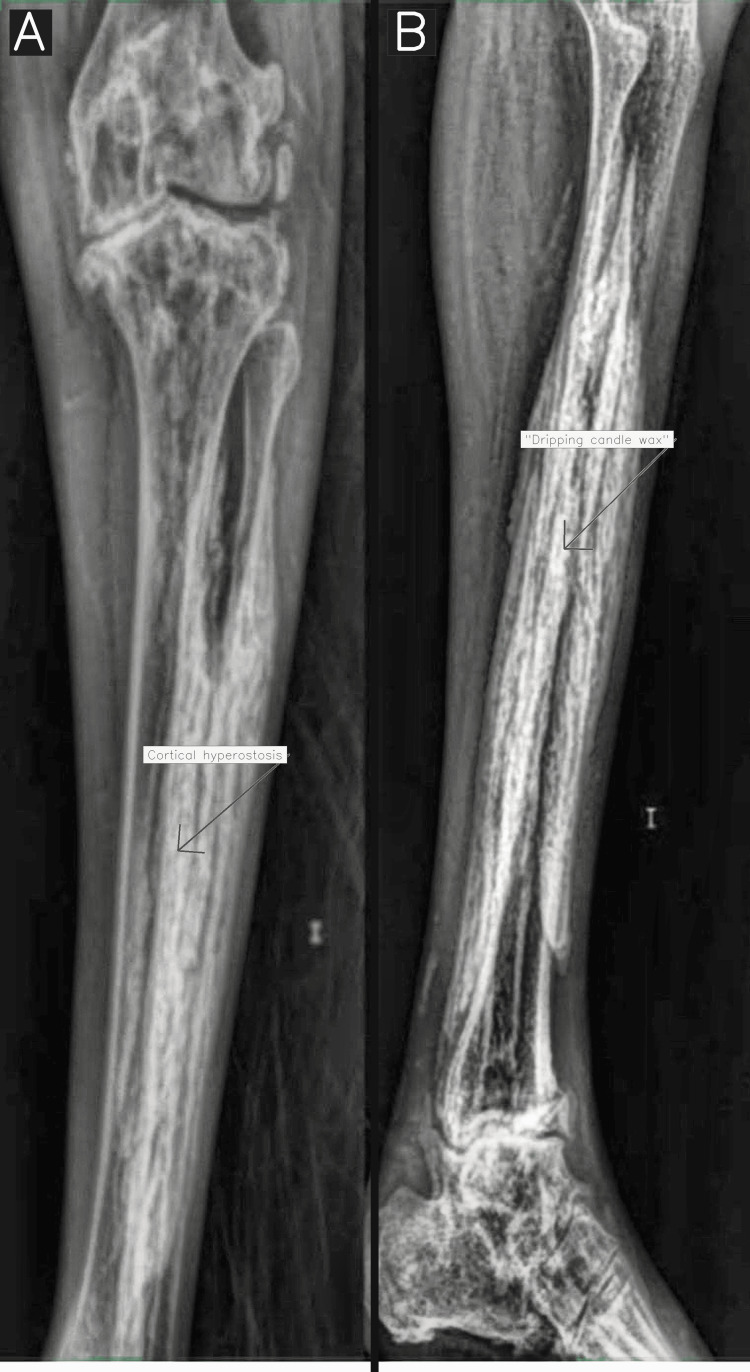
Plain radiographs of the left leg demonstrating melorheostotic hyperostosis. (A) Anteroposterior view showing eccentric periosteal and endosteal cortical thickening of the tibia and fibula with the characteristic “dripping candle wax” appearance. (B) Lateral view confirming the irregular longitudinal hyperostosis along the cortical surfaces.

Conventional radiographs of the left leg, shown in Figure 1A, 1B, revealed focal areas of irregular, dense radiopacity distributed in an eccentric pattern along both the periosteal and endosteal cortical surfaces of the left tibia and fibula. This appearance corresponded to the characteristic “dripping candle wax” sign, a pathognomonic feature of melorheostosis [4]. No articular involvement or perilesional fractures were identified. Computed tomography was not performed, as the characteristic radiographic findings in conjunction with MRI provided sufficient diagnostic information. Additionally, cost-accessibility considerations and the absence of diagnostic ambiguity following multimodal evaluation supported this decision.

Biochemical workup was unremarkable. Serum calcium, phosphorus, total and bone-specific alkaline phosphatase, C-reactive protein, and erythrocyte sedimentation rate all fell within normal reference ranges, which is consistent with the typical laboratory profile of this condition.

**Figure 2:**
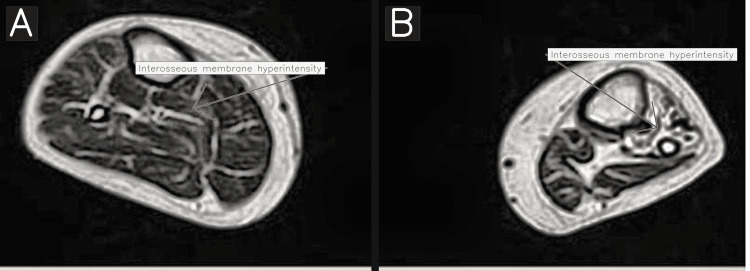
Axial MRI sequences of the left leg showing soft tissue involvement. (A) Proximal cross-section demonstrating hypotrophic anterior and lateral compartment musculature with hypointense signal areas. (B) Distal cross-section revealing hypointense regions adjacent to the fibula, consistent with mineralization within the peroneal and posterior tibial muscles.

Magnetic resonance imaging (MRI) of the left leg, illustrated in Figure 2A, 2B, demonstrated hypotrophic musculature in the anterior and lateral compartments. Hypointense signal areas on T1- and T2-weighted sequences were identified within the posterior tibial, peroneus longus, and soleus muscles in proximity to the fibula, reflecting mineralization and fibrous replacement of the soft tissues surrounding the hyperostotic bone.

**Figure 3:**
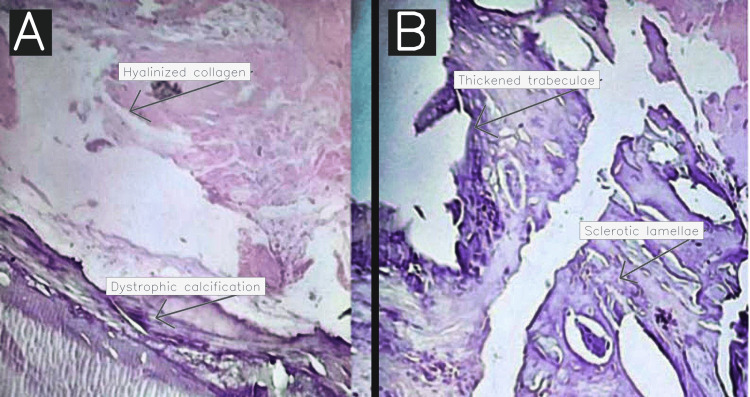
Histopathological findings of the tibial biopsy (H&E stain). (A) Low-power view showing hyalinized collagen fibers and dystrophic calcifications within the bone matrix. (B) Higher-magnification view demonstrating thickened, irregular sclerotic lamellae with increased osteoblastic activity and disorganized lamellar architecture.​​​​​​​​​​​​​​​​

A percutaneous biopsy of the tibial lesion was subsequently performed. Histopathological analysis, depicted in Figure 3A, 3B, identified disorganized lamellar bone with thickened, irregular trabeculae, increased osteoblastic activity, hyalinized collagen fibers, and scattered foci of dystrophic calcification. No special stains or ancillary immunohistochemical techniques were employed, as hematoxylin and eosin-stained sections provided sufficient morphological evidence to establish the diagnosis and confidently exclude malignant or infectious etiologies.

These findings were consistent with the known microscopic architecture of melorheostosis, ruling out malignant or infectious etiologies.

Based on the integration of clinical, radiological, laboratory, and histopathological data, a definitive diagnosis of monomelic melorheostosis involving the left tibia and fibula was established. Given the patient’s functional impairment and pain burden, a conservative multimodal treatment plan was initiated. This included celecoxib 100 mg daily for nociceptive pain control, in conjunction with a supervised physical therapy program emphasizing passive range-of-motion exercises, progressive stretching of the posterior compartment, and soft tissue mobilization techniques.

At initial presentation, the patient reported a pain score of 4/10 on the Visual Analogue Scale (VAS). Given the patient’s functional impairment and pain burden, a conservative multimodal treatment plan was initiated, including celecoxib 100 mg daily for nociceptive pain control, in conjunction with a supervised physical therapy program emphasizing passive range-of-motion exercises, progressive stretching of the posterior compartment, and soft tissue mobilization techniques. At six-month follow-up, the patient demonstrated a favorable clinical response, with pain declining to 1/10 on the VAS and substantial improvement in active range of motion at the ankle. No surgical intervention was deemed necessary. The patient remains under close outpatient surveillance with periodic imaging to monitor for disease progression or the development of joint contractures.

## Discussion

The present case illustrates several atypical features of melorheostosis that merit discussion in the context of the existing literature. The late onset of symptoms in a fifth-decade female patient, the monomelic distribution confined to the left tibia and fibula, and the favorable response to conservative management collectively highlight the clinical and therapeutic heterogeneity of this condition.

Melorheostosis ranks among the rarest musculoskeletal disorders documented in the literature, with a global prevalence estimated at fewer than one case per million individuals. Nearly half of all diagnosed patients receive their diagnosis before reaching the age of 20, and this early-onset subset tends to experience a more rapidly progressive clinical trajectory. In contrast, individuals who develop the condition later in life, such as the patient described in this report, typically follow a more protracted and indolent course. Although the condition has generally been considered to affect both sexes equally, certain series have reported a female predominance with ratios approaching 4:1 [4].

The disorder most frequently involves the diaphysis and epiphysis of long bones, and axial skeleton involvement is considerably less common. Depending on the extent and anatomical pattern of skeletal involvement, melorheostosis is subclassified as monostotic (a single bone), polyostotic (multiple bones), monomelic (a single limb), or hemimelic (one half of the body); the monomelic variant constitutes the most frequently encountered presentation. Additionally, melorheostosis may co-occur with other sclerosing bone conditions, a configuration referred to as overlap syndrome [5].

The differential diagnosis of melorheostosis encompasses several sclerosing bone conditions with overlapping radiological features, including osteopoikilosis, osteopathia striata, chronic osteomyelitis, and low-grade parosteal osteosarcoma. Osteopoikilosis characteristically presents as symmetric, oval sclerotic foci concentrated in the epiphyses, without the flowing cortical hyperostosis seen in melorheostosis. Osteopathia striata manifests as bilateral longitudinal metaphyseal striations, in contrast to the unilateral sclerotomal distribution of melorheostosis. Chronic osteomyelitis is distinguished by an infectious clinical context, elevated inflammatory markers, and periosteal reaction with adjacent soft tissue edema. Low-grade parosteal osteosarcoma lacks the sclerotomal distribution and typically demonstrates cortical destruction on histopathological examination. In the present case, the combination of strictly normal laboratory values, pathognomonic monomelic radiographic pattern, and benign histopathological architecture collectively excluded these entities and supported the definitive diagnosis of melorheostosis.

Pathophysiology

Substantial progress in understanding the molecular underpinnings of melorheostosis has emerged in recent years. Somatic mutations in the MAP2K1 gene have been identified in patients exhibiting the cortical form of the disease. These mutations disrupt normal bone microarchitecture and provoke a periosteal reaction histologically reminiscent of osteomyelitis or traumatic injury, ultimately generating the characteristic wavy radiological appearance. In parallel, alterations in the SMAD3 gene have been implicated in the endosteal variant, where they appear to potentiate signaling through the TGF-β/SMAD pathway and promote abnormal bone deposition [6].

The overarching pathogenetic mechanism is thought to involve somatic mosaicism, which accounts for the asymmetric, segmental, and non-hereditary distribution of lesions. Mutations involving the LEMD3 gene have also been reported, and various developmental, ischemic, and infectious triggers have been proposed as contributing factors, frequently in association with osteopoikilosis or LEMD3 variants [7].

Diagnosis

Establishing a diagnosis of melorheostosis requires synthesizing clinical manifestations with a multimodal imaging evaluation and, when necessary, histopathological confirmation. Patients most commonly present with limb deformity, joint or bone pain, stiffness, and restricted range of motion; pain and reduced mobility represent the cardinal complaints [8]. Soft tissue involvement is diverse and may encompass subcutaneous fibrosis, cutaneous erythema, scleroderma-like linear plaques, ectopic ossification, edema, hypertrichosis, fibromas, fibrolipomas, capillary hemangiomas, lymphangiectasia, and arterial aneurysms, all of which can precipitate joint contractures and further limit functional capacity [9]. Retraction of ligaments and tendons secondary to fibrosis is not infrequent, and foot deformities including equinovarus, valgus, and varus configurations have been well described [10]. Early onset of symptoms and multimember involvement are considered predictors of a more complicated disease course.

Radiographically, melorheostosis predominantly involves the long bones of the lower extremities, with comparatively less frequent involvement of the short bones of the hands and feet, and rare craniofacial manifestations. The classic plain radiograph demonstrates dense, irregular, eccentric hyperostosis along the periosteal and endosteal cortical surfaces of one or more contiguous bones, producing the iconic “melted candle wax” pattern [11,12]. Beyond this classic form, three additional radiographic variants have been described: an osteoma-like pattern confined to the endosteal surface along the longitudinal bone axis; a striated osteopathy-like pattern featuring dense unilateral hyperostotic striations near the inner cortical surface of two or more bones; and a myositis ossificans-like pattern involving two or more unilateral regions, with or without intraosseous hyperostosis [13].

Computed tomography offers superior resolution for quantifying the degree of cortical sclerosis and medullary canal compromise compared to plain radiography. On MRI, hyperostotic areas yield characteristically low signal intensity on both T1- and T2-weighted sequences, with variable gadolinium enhancement; soft tissue involvement may produce heterogeneous signal corresponding to zones of mineralization intermixed with adipose and fibrovascular components [14]. Histopathologically, the affected bone displays disorganized osteoid formation, increased angiogenesis, thickened and irregular lamellar trabeculae, and elevated osteoblastic activity. Cortical thickening composed of primitive Haversian systems partially obliterated by sclerotic lamellae is the predominant microscopic finding. Associated scleroderma-like skin lesions show collagen that differs from classic systemic scleroderma and has been specifically designated as linear melorheostotic scleroderma [15].

Standard laboratory evaluation in melorheostosis characteristically yields normal serum levels of calcium, phosphorus, total alkaline phosphatase, and bone-specific alkaline phosphatase, except in the context of recent fracture or stress fracture. Markers of bone turnover - including serum type 1 procollagen N-propeptide, serum beta-C-terminal telopeptide (beta-CTx), and urinary N-terminal telopeptide - have likewise been found within normal limits in the available literature [16].

Treatment

The principal sources of morbidity in melorheostosis are chronic pain, progressive joint contractures, restricted mobility, limb length inequality, and skeletal deformity. Historically, management relied predominantly on surgical techniques such as tendon lengthening, excision of soft tissue masses, contracture release, and corrective osteotomy; however, recurrence following these procedures is well documented, and functional complications arise in up to 54% of surgically treated cases [17]. No validated clinical guidelines or controlled trials currently exist to direct medical therapy for painful hyperostosis in this condition.

Pain in melorheostosis is multimechanistic and may be nociceptive, neuropathic, or skeletal in origin. Nociceptive pain driven by soft tissue calcification surrounding hyperostotic bone frequently responds to structured physical therapy, including progressive stretching, tissue mobilization, and analgesics. Neuropathic pain attributable to bony compression of adjacent neural structures may benefit from oral neuropathic agents, decompressive osteotomy, perineural injection therapies, or spinal cord stimulation. Skeletal pain related to elevated intraosseous pressure, heightened vascularization, and increased osteoclastic activity may require additional targeted interventions [18].

Physical therapy and symptomatic pharmacological management remain the most robustly supported treatment pillars for melorheostosis. Physical modalities, employing thermal, electrical, or pressure-based stimuli, may modulate pain signaling at peripheral, spinal, and supraspinal levels depending on the parameters applied. Passive range of motion exercises, splinting, and orthotics are particularly valuable for preventing contractures or arresting their progression during early stages; advanced contractures may become fixed and require surgical correction. Preventive stretching regimens should incorporate holds of approximately 15 seconds with 10 to 15 repetitions per session, individualized to the affected anatomical regions [19]. Antiresorptive therapy with nitrogen-containing bisphosphonates has been explored given their capacity to inhibit osteoclast-mediated bone resorption through direct and indirect actions on osteoblasts and macrophages. Published case reports have documented symptomatic improvement with oral pamidronate (30 mg daily for six days) and more pronounced benefit, including reductions in pain intensity, local warmth, and lesion size with intravenous zoledronic acid (5 mg), which is hypothesized to additionally reduce bone vascularization and inhibit angiogenesis beyond its primary antiresorptive action [20].

Emerging targeted therapies represent a promising future direction for melorheostosis management. Given the established role of MAP2K1 somatic mutations in the cortical form of the disease, MEK inhibitors have been proposed as potential disease-modifying agents. Although clinical evidence remains limited to preclinical and early-phase investigations, these molecules may ultimately offer therapeutic options beyond current symptomatic management, particularly in patients with refractory pain or progressive deformity.

Optimal care of patients with melorheostosis requires a coordinated multidisciplinary team encompassing orthopedic surgery, physical medicine and rehabilitation, and pain management specialists. A comprehensive, individualized treatment strategy that integrates analgesic optimization, structured rehabilitation, and judicious use of antiresorptive or anti-inflammatory agents offers the most favorable outcomes currently achievable. Advancing molecular research aimed at characterizing the genetic and developmental contributors to this disease holds promise for the eventual development of disease-modifying targeted therapies.

## Conclusions

This case contributes to the limited published literature on melorheostosis by documenting an atypical late-onset, monomelic presentation in a fifth-decade female patient with involvement of the left tibia and fibula. The diagnostic integration of pathognomonic radiographic findings, normal laboratory parameters, and compatible histopathological features allowed definitive diagnosis without advanced molecular testing, underscoring the continued relevance of systematic multimodal clinical reasoning in rare bone dysplasias.

The favorable six-month outcome achieved through conservative management combining celecoxib and structured physical therapy reinforces that surgical intervention should be reserved for refractory or advanced cases, given its documented recurrence and functional complication rates. Clinicians encountering unexplained cortical hyperostosis with sclerotomal distribution should maintain a high index of suspicion for melorheostosis, even in atypical demographic presentations. As molecular characterization advances, targeted therapies such as MEK inhibitors may emerge as viable disease-modifying options, shifting management from purely symptomatic approaches toward precision medicine strategies.
